# In Situ Measurement and Reconstruction Technology of Cylindrical Shape of High-Precision Mandrel

**DOI:** 10.3390/mi14061240

**Published:** 2023-06-12

**Authors:** Hanwei Xu, Zizhou Sun, Yifan Dai, Chaoliang Guan, Hao Hu

**Affiliations:** 1 College of Intelligent Science and Technology, National University of Defense Technology, Changsha 410073, China; xuhanwei21@nudt.edu.cn (H.X.); 18302996932@163.com (Z.S.); chlguan@nudt.edu.cn (C.G.); ting_hh@139.com (H.H.); 2 Hunan Key Laboratory of Ultra-Precision Machining Technology, Changsha 410073, China; 3 Laboratory of Science and Technology on Integrated Logistics Support, National University of Defense Technology, Changsha 410073, China; 4 Nanhu Laser Laboratory, National University of Defense Technology, Changsha 410073, China

**Keywords:** in situ measurement and reconstruction technology, cylindrical shape, three-point method, high-precision mandrel

## Abstract

The technology of in situ measurement of cylindrical shapes is an important means of improving the surface machining accuracy of cylindrical workpieces. As a method of cylindricity measurement, the principle of the three-point method has not been fully studied and applied, so it is seldom used in the field of high-precision cylindrical topography measurement. Since the three-point method has the advantages of a simpler measurement structure and smaller system error compared with other multi-point methods, the research on it is still of great significance. Based on the existing research results of the three-point method, this paper proposes the in situ measurement and reconstruction technology of the cylindrical shape of a high-precision mandrel by means of a three-point method. The principle of the technology is deduced in detail and an in situ measurement and reconstruction system is built to carry out the experiments. Experiment results are verified using a commercial roundness meter and the deviation of cylindricity measurement results is 10 nm, which is 2.56% of the measurement results of commercial roundness meters. This paper also discusses the advantages and application prospects of the proposed technology.

## 1. Introduction

High-precision cylindrical parts have been widely used in the fields of deep space exploration and ultra-precision manufacturing. How to improve the surface machining accuracy of cylindrical parts has become an obstacle restricting the development of the industry. At present, there are mainly three methods to improve the surface machining accuracy of cylindrical parts: The first method is to use ultra-precision cylindrical grinders or lathes. With the help of the precision of the machine tool itself, the high precision of the machined parts is guaranteed. The machine tools used in this method are generally extremely expensive and have strict requirements for the machining environment. The second method is to use off-position measurement [1] and compensation machining to improve the accuracy of the surface shape. Compared with the first method, this method has a lower cost, but repeated part clamping will introduce secondary clamping errors and reduce processing efficiency. At the same time, the environment of off-position measurement is generally different from the machining environment, which may easily cause the measurement results to not accurately guide the compensation machining. The third method is to use the method of in situ measurement [2,3,4] and compensation machining [5,6] to improve the accuracy of the surface shape. This method not only reduces the requirements on the performance of the machine tool but also avoids the impact of the secondary clamping error and environmental changes. Based on the advantages of the third method, it has been one of the hot spots in the field of advanced manufacturing technology to apply this method to the in situ measurement of cylindrical shape.

K. Stepien [2] systematically introduced three methods of cylindrical shape measurement, including the multi-probe method, the V-block method, and the Articulated Arm Coordinate Measuring Machines. The advantages and disadvantages of the three methods are compared in the paper, and it is considered that the multi-probe method is the most suitable method for carrying out high-precision in situ measurement of small-sized cylindrical workpieces. The multi-probe method uses multiple sensors to collect the cylindrical shape data synchronously and integrates the data for error separation. Multi-probe methods mainly include the three-point method, four-point method, and five-point method.

The traditional three-point method for measuring cylindricity was first extended from the three-point method for error separation of roundness. This method assumes that the axis of the cylindrical workpiece being measured is an ideal straight line. In fact, the axis of the cylindrical workpiece is not a straight line, so the measurement results of the traditional three-point method are not accurate. However, this method is still very effective for measuring large cylinders or where the accuracy requirement is not high. Both S. Ozono [7] and B. Muralikrishnan [8] conducted in-depth research on the three-point method and believed that the three-point method has the problem of first-order harmonic suppression, so the extraction of the least square circle center of the cross section cannot be realized. Kakino [9] and Lei [10], respectively, introduced the improved method of the three-point method, which can realize the measurement of the roundness of each cross section, the straightness of the axis, and the deviation of the average radius of each cross section. They used a mathematical algorithm to avoid the problem of first harmonic suppression in the three-point method, and the position of the least square center can be obtained accurately. Their research achievement is great progress on the three-point measurement of cylindricity. However, the influence of zeroing error on the measurement results has not been fully considered. The existence of zeroing error will lead to inaccurate measurement results of the average radius of each cross section, which will further affect the accuracy of cylindrical shape reconstruction.

Nyberg [11] proposed a four-point method for cylindricity error separation technology. Three sensors are arranged on the same cross section of a cylindrical workpiece to measure the roundness according to the three-point method error separation technology [12,13]. Two sensors are arranged on the same busbar to realize the measurement of busbar straightness according to the sequential-two-points method [14,15]. Of all the sensors, one is shared. Nyberg demonstrated the effectiveness of this approach by building a measurement setup. However, the four-point method still has defects in principle; that is, the sequential-two-points method cannot realize the separation of the tilting motion error of the busbar. Katsuyuki [16] established a five-point scanning system. The five scanning sensors are all located on the same spiral line on the surface of the cylinder. The rotation of the cylinder is synchronized with the motion of the sensors so that the cylinder profile measurement is transformed into a one-dimensional straightness measurement. The measurement results of this system are compared with those of commercial roundness meters, and this proves the feasibility of the five-point method. However, the multi-point straightness error separation technology based on discrete Fourier transform assumes that the straightness profile is end to end. It is impossible to realize this assumption with the actual spiral profile on a cylinder. Therefore, the straightness error separation technology based on the discrete Fourier transform is still insufficient in principle to measure and reconstruct the spiral profile on the cylinder. On the basis of summarizing the previous research results, Liu put forward a new four-point method [17] and another five-point method [18], respectively. However, its research object is large cylindrical workpieces, such as large cylindrical parts used in papermaking or other fields, so the requirements for measurement accuracy are relatively low. At the same time, the four-point cylindricity measurement method has the problem of poor anti-interference ability, and the five-point cylindricity measurement method also has the defect of complex structure. Therefore, these two methods proposed by Liu are not suitable for in situ measurement of small-sized and high-precision cylindrical parts.

With the research of the multi-point method entering the bottleneck, the high-precision in situ measurement method of cylindricity begins to develop towards hybrid methods [19] and optical measurement methods [20,21,22].

In general, the traditional three-point method can only realize the in situ measurement of cylindrical workpieces with low surface accuracy. Kakino and Lei’s research results further improved the function of the three-point method, but the influence of zeroing error on error separation was ignored. Due to the influence of sensor size, the measurement structure of the four-point method and the five-point method is complicated, which makes it not suitable for measuring small-size workpieces. In addition, the four-point method has errors in principle and the five-point method has poor anti-interference ability, so it is difficult to realize high-precision cylindrical shape reconstruction. Therefore, the three-point method is the most suitable method for measuring small-sized and high-precision cylindrical workpieces. Based on the current research status, the three-point method still leaves room for improvement.

In this paper, according to the elements of cylindrical shape reconstruction defined in [23,24], the cylindricity measurement method of the three-point method is further optimized. The in situ measurement and reconstruction technology of the cylindrical shape of the high-precision mandrel by the three-point method is put forward in this paper. Mathematical means are used to identify the data of axis straightness and average radius of each cross section. At the same time, the influence of the first harmonic components of spindle radial errors, zeroing errors of sensors, and clamping eccentricity errors of the workpiece on error separation accuracy are avoided.

## 2. Establishment of In Situ Measurement Model of High-Precision Mandrel

**Figure 1:**
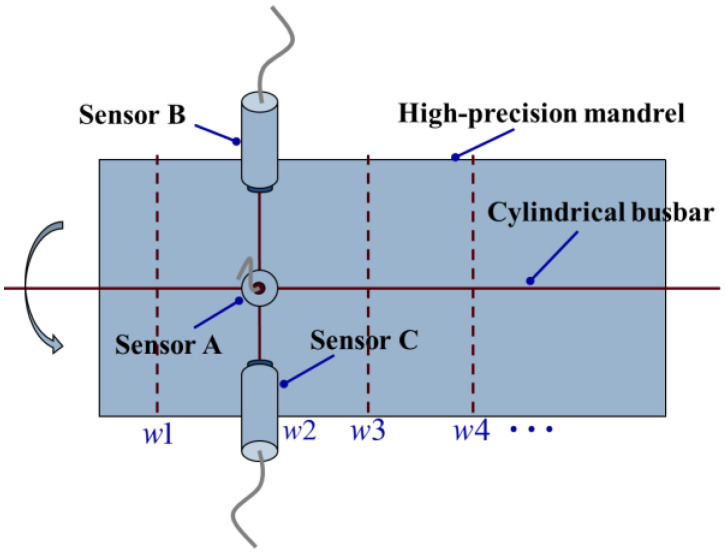
Schematic diagram of cylindrical shape measurement of high-precision mandrel.

In this paper, the research object is a high-precision mandrel. The placement position of the sensors of the three-point method is shown in Figure 1. Sensors A, B, and C are arranged on the same cross section of the high-precision mandrel. Three sensors need to be fixed on the slide by an integrally processed fixture, moving with the slide along the cylindrical busbar of the high-precision mandrel to achieve the purpose of measuring different cross sections. In Figure 1, w1,w2,w3,w4,⋯ are the cross sections of the high-precision mandrel to be measured. According to the three-point roundness error separation technology, the in situ measurement of roundness can be realized by using the three sensors, while the radial error and the least square circle center of each cross section can be realized through harmonic analysis. With radial error known, the average radius of each cross section can be separated from the data of Sensor A. Thus, the three elements of reconstructing the cylindrical shape of the high-precision mandrel can be obtained, and the following will give the specific theoretical analysis process.

### 2.1. Establishment of In Situ Measurement Coordinate System

**Figure 2:**
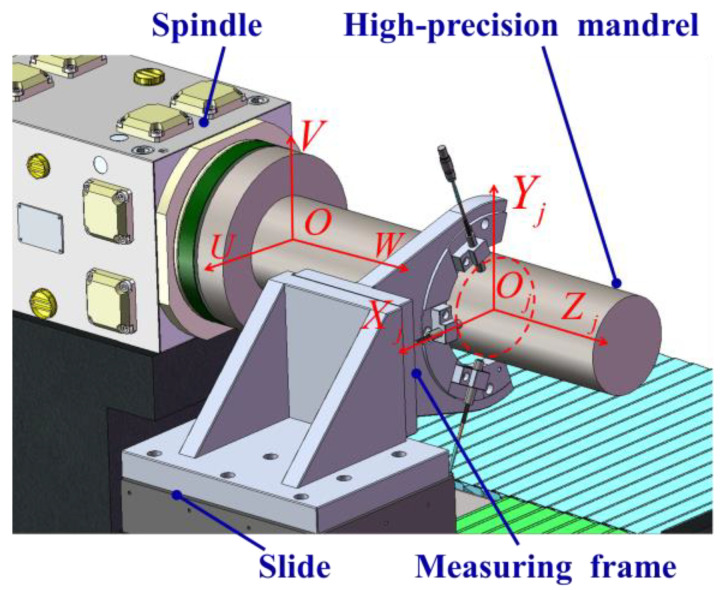
In situ measurement device model coordinate systems.

As shown in Figure 2, the model of the in situ measurement device mainly consists of a spindle, slide, high-precision mandrel, and measuring frame. The measuring frame is composed of sensors and measuring fixtures. The high-precision mandrel is fixed on the spindle of the ultra-precision lathe and can rotate at a constant speed with the spindle. The measuring frame is fixed on the slide and can move parallel to the spindle axis. To describe the error relationship of in situ measurements more clearly, coordinate systems are established as shown in Figure 2. Firstly, take the intersection point of the average line of the rotation axis and the spindle end face as the origin O and establish the absolute coordinate system. The coordinate axis U and V are located horizontally and vertically, respectively, while the coordinate axis W coincides with the average line of the axis of rotation of the spindle. Secondly, taking the intersection of the axes of the three sensors as the origin $O_{j}$, the measuring coordinate system of cross section j is established. The coordinate axis $X_{j}$, $Y_{j}$ and $Z_{j}$ are parallel to the coordinate axis U, V and W of the absolute coordinate system, respectively. The number j can be taken as 1,2,⋯M, and M is the total number of the measured cross sections.

### 2.2. Establishment of High-Precision Mandrel Mathematical Model

The profile of the cross section of the high-precision mandrel in the rotation will exhibit deterministic periodic characteristics, which can be expanded in the Fourier series as

(1) $$V(\theta )=A_{0}+\sum_{k=1}^{\infty }\left(A_{k}\cos k\theta +B_{k}\sin k\theta \right)$$

where θ is the angle value corresponding to each point on the cross section; $A_{0}$ is the average radius of the cross section; k stands for harmonic order; and $A_{k}$ and $B_{k}$ represent the sine and cosine Fourier coefficients of the roundness error, respectively.

The high-precision mandrel can be regarded as a continuum with numerous circular cross sections. Therefore, a similar Fourier expansion can be used to represent the cylindrical shape of the high-precision mandrel. It should be noted that $A_{k}$ and $B_{k}$ are functions related to the number of each cross section, so the cylindrical shape $V_{j}(\theta )$ of the high-precision mandrel can be expressed as

(2) $$V_{j}(\theta )=A_{0j}+\sum_{k=1}^{\infty }\left(A_{kj}\cos k\theta +B_{kj}\sin k\theta \right)$$

where j is the number of each cross section; $A_{0j}$ is the average radius of the cross section j; and $A_{kj}$ and $B_{kj}$ represent the sine and cosine Fourier coefficients of the roundness error of the cross section j, respectively.

In order to describe each error component in $V_{j}(\theta )$ more directly, use the orthogonal polynomials to fit $A_{0j}$, $A_{kj}$, and $B_{kj}$ as

(3) $$\left\{\begin{array}{l} A_{0j}=\sum_{l=0}a_{l0}\cdot P_{l}(w_{j})\\ A_{kj}=\sum_{l=0}a_{lk}\cdot P_{l}(w_{j})\\ B_{kj}=\sum_{l=0}b_{lk}\cdot P_{l}(w_{j}) \end{array}\right.$$

Among them, $P_{l}(w_{j})$ is an orthogonal polynomial; l is the order of orthogonal polynomial; $w_{j}$ is the W coordinate value of each section in the absolute coordinate system; and $a_{l0}$, $a_{lk}$, and $b_{lk}$ are coefficients.

$P_{l}(w_{j})$ can be taken as a Legendre polynomial, and its expression is

(4) $$\left\{\begin{array}{l} P_{0}(w_{j})=1\\ P_{1}(w_{j})=w_{j}\\ \begin{array}{cc} \begin{array}{cccc} & & \vdots & \end{array} & \end{array}\\ P_{l}(w_{j})=\frac{1}{2^{l}l!}\cdot \frac{d^{l}}{dw_{j}^{l}}[{\left(w_{j}^{2}-1\right)}^{l}] \end{array}\right.$$

Substituting Equations (3) and (4) with Equation (2), we can calculate

(5) $$\begin{array}{l} V_{j}(\theta )=A_{0}{}_{j}+\sum_{k=1}\sum_{l=0}a_{l}{}_{k}P_{l}(w_{j})\cos k\theta +\sum_{k=1}\sum_{l=0}b_{l}{}_{k}P_{l}(w_{j})\sin k\theta \\ =A_{0j}+(a_{01}\cos\theta +b_{01}\sin\theta )+(a_{11}w_{j}\cos\theta +b_{11}w_{j}\sin\theta )\\ +\sum_{l=2}\left(a_{l1}\cos\theta +b_{l1}\sin\theta )P_{l}(w_{j}\right)+\sum_{k=2}\left(A_{kj}\cos k\theta +B_{kj}\sin k\theta \right) \end{array}$$

The right side of the equal sign in Equation (5) can be regarded as the sum of five terms. Among them, the first term is only related to j, so it is the error of the radius of each cross section. The second term has nothing to do with j, which means that the centers of each cross section are on a straight line, and the offset between the straight line and datum axis is $\sqrt{a_{01}{}^{2}+b_{01}{}^{2}}$. The third term indicates that the center of each cross section is on a straight line with an inclination of $\left(a_{11},b_{11}\right)$ to the datum axis. The fourth term represents the location of the center of each cross section. The fifth term represents the roundness error of each cross section. It can be known that the second and third terms only indicate the relative position between the axis of the high-precision mandrel and the datum axis. Only the first, fourth, and fifth terms determine the cylindrical shape of the high-precision mandrel, which is consistent with the statement in reference [23,24].

Based on the analysis above, Equation (5) can be reduced to

(6) $$\begin{array}{l} V_{j}(\theta )=A_{0j}+\sum_{l=2}\left(a_{l1}\cos\theta +b_{l1}\sin\theta )P_{l}(w_{j}\right)\\ +\sum_{k=2}\left(A_{kj}\cos k\theta +B_{kj}\sin k\theta \right) \end{array}$$

Among them, $A_{0j}$ is the variation or relative radius of the average radius of cross section j; $\sum_{l=2}\left(a_{l1}\cos\theta +b_{l1}\sin\theta )P_{l}(w_{j}\right)$ is the bending of the central axis at the cross section j; and $\sum_{k=2}\left(A_{kj}\cos k\theta +B_{kj}\sin k\theta \right)$ is the roundness error of cross section j.

For the convenience of subsequent expression, Equation (6) can be abbreviated as

(7) $$V_{j}(i)=A_{0j}+R_{1j}+R_{2j}$$

Among them, $R_{1j}$ represents $\sum_{l=2}\left(a_{l1}\cos\theta +b_{l1}\sin\theta )P_{l}(w_{j}\right)$, while $R_{2j}$ represents $\sum_{k=2}\left(A_{kj}\cos k\theta +B_{kj}\sin k\theta \right)$.

**Figure 3:**
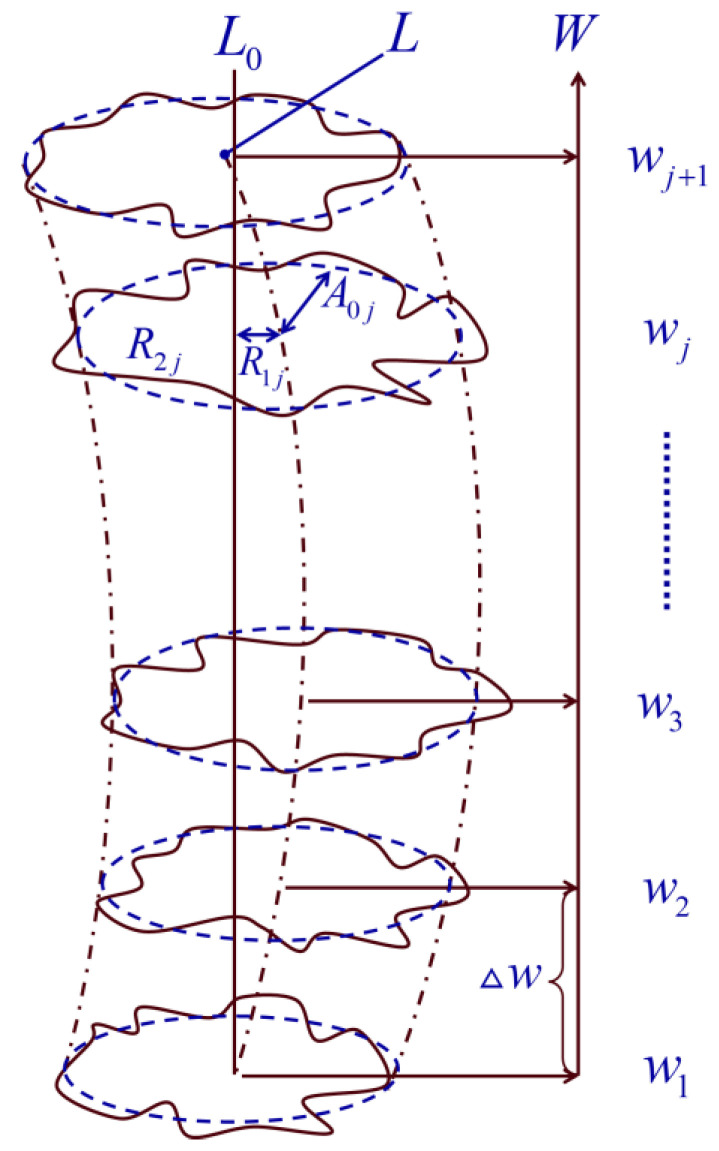
Schematic diagram of cylindrical shape.

Thus, the shape of the high-precision mandrel can be fitted from multiple cross sections, as shown in Figure 3. $w_{1},w_{2},\cdots w_{j},w_{j+1}$, respectively, represent the W coordinate values corresponding to different cross sections. $L_{0}$ is the ideal axis. L is the actual axis.

### 2.3. Algorithm for In Situ Measurement of Cylindrical Shape by Means of the Three-Point Method

#### 2.3.1. Analysis of In Situ Measurement Model

**Figure 4:**
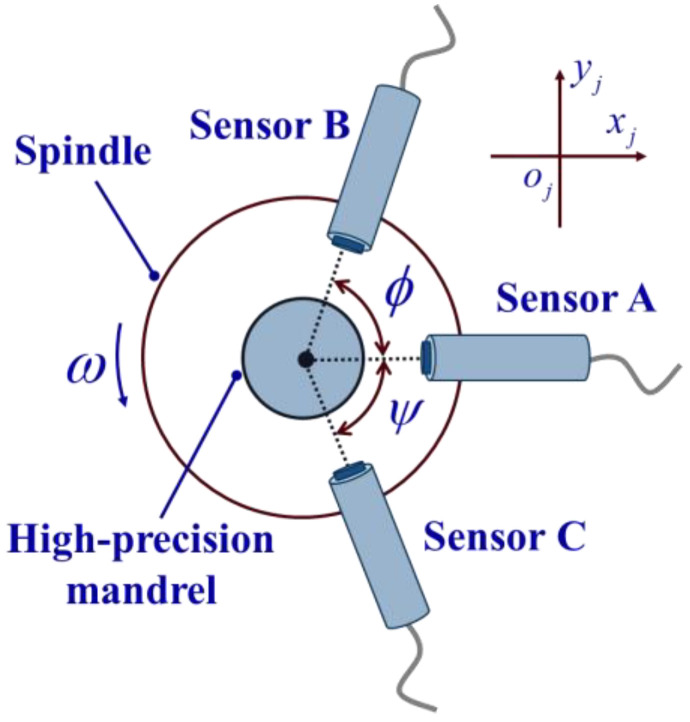
Schematic diagram of three-point roundness measurement.

In the measurement coordinate system $O_{j}-X_{j}Y_{j}Z_{j}$ of cross section j, an in situ measurement and error separation model is established, as shown in Figure 4. The three sensors are placed around the cross section for data acquisition synchronously. The determination of the installation angle [25,26,27] requires specific calculations to avoid harmonic suppression. In this paper, $\phi =m_{1}\times 2\pi /N$, $\psi =m_{2}\times 2\pi /N$, $m_{1}=100$, $m_{2}=91$, and the number of sampling points N for each circle is 512. The data sequences collected by Sensors A, B, and C are denoted as $r_{Aj}(i)$, $r_{Bj}(i)$, and $r_{Cj}(i)$, i=0,1,2,⋯,N−1.

In the measurement coordinate system, the data collected by the sensors include not only the profile data of the measured cross section but also the radial error of the spindle, the installation eccentricity error of the high-precision mandrel, the zeroing errors of the sensors, and the errors caused by environmental noise. Separating the various error components from the data collected by the sensors is the key to illuminating the reconstructed cylindrical shape. The following will continue to introduce the separation method of each element required for the reconstruction of the cylindrical shape.

#### 2.3.2. Calculation of $R_{2j}$

Although the periodicity of radial error of ultra-precision spindle has not been proved, it is an indisputable fact that the higher the radial accuracy is, the better the repeatability of rotation error is. Therefore, both the radial error and the eccentricity error are periodic error components in the data collected by the sensors, so the eccentricity error can be regarded as a part of the radial error data, which can be recorded as $S_{j}(i)$. The roundness profile of the cross section can be represented by $V_{j}(i)$.

After eliminating the noise error in the original data of the sensors, the data collected by three sensors can be expressed as

(8) $$r_{Aj}(i)=V_{j}(i)+S_{j}(i)\cos(2\pi i/N)+d_{A}$$

(9) $$\begin{array}{l} r_{Bj}(i)=V_{j}(i-m_{1})+S_{j}(i)\cos(2\pi i/N)\cos(\phi )\\ +S_{j}(i)\sin(2\pi i/N)\sin(\phi )+d_{B} \end{array}$$

(10) $$\begin{array}{l} r_{Cj}(i)=V_{j}(i+m_{2})+S_{j}(i)\cos(2\pi i/N)\cos(\psi )\\ -S_{j}(i)\sin(2\pi i/N)\sin(\psi )+d_{C} \end{array}$$

Among them, $m_{1}$ and $m_{2}$ are the sampling points covered by the angle ϕ and ψ between the sensors; $d_{A}$, $d_{B}$, and $d_{C}$ are the zeroing errors of Sensors A, B, and C, respectively.

Since the three-point roundness error separation method is a classical error separation method, only the conclusion is given here. According to reference [12], the weighted sum of the sampling data of the three sensors can be calculated by selecting the appropriate parameter a and b.

(11) $$\begin{array}{l} r_{j}(i)=r_{Aj}(i)+ar_{Bj}(i)+br_{Cj}(i)\\ =(1+a+b)A_{0j}+(d_{A}+a\times d_{B}+b\times d_{C})\\ +\sum_{k=2}^{\infty }\left[A_{kj}\cos(2\pi ki/N)+B_{kj}\sin(2\pi ki/N)\right]\\ +a\sum_{k=2}^{\infty }\left\{A_{kj}\cos[2\pi k(i-m_{1})/N]+B_{kj}\sin[2\pi k(i-m_{1})/N]\right\}\\ +b\sum_{k=2}^{\infty }\left\{A_{kj}\cos[2\pi k(i+m_{2})/N]+B_{kj}\sin[2\pi k(i+m_{2})/N]\right\} \end{array}$$

At the same time, $r_{j}(i)$ can be directly changed into the following Fourier series:

(12) $$r_{j}(i)=F_{0j}+\sum_{k=1}^{\infty }\left[F_{kj}\cos(2\pi ki/N)+G_{kj}\sin(2\pi ki/N)\right]$$

The roundness error of the cross section can be obtained by solving Equations (11) and (12) simultaneously:

(13) $$R_{2j}=\sum_{k=2}^{\infty }\left[A_{kj}\cos(2\pi ki/N)+B_{kj}\sin(2\pi ki/N)\right]$$

At the same time, the zero-order harmonics of Equations (11) and (12) are also equal to

(14) $$(1+a+b)A_{0j}+(d_{A}+a\times d_{B}+b\times d_{C})=F_{0j}$$

Since $d_{A}$, $d_{B}$, and $d_{C}$ are unknown, $A_{0j}$ cannot be solved. Due to the problem of first harmonic suppression, the least square center of cross section j cannot be obtained either.

#### 2.3.3. Calculation of $R_{1j}$

Since $A_{0j}$ cannot be calculated directly by the three-point error separation technology, it is advisable to eliminate the zero-order harmonic components from the raw data. As a result, the interference of zeroing error on error separation can be avoided.

Without considering the zero-order harmonic components, the profile of cross section j can be expressed as

(15) $$\begin{array}{l} V_{j}^{\prime }(i)=V_{j}(i)-A_{0j}=R_{1j}(i)+R_{2j}(i)\\ =A_{1j}\cos(2\pi i/N)+B_{1j}\sin(2\pi i/N)+R_{2j}(i) \end{array}$$

Since $R_{2j}(i)$ and $V_{j}^{\prime }(i)$ are periodic sequences, it can be known according to Equation (15)

(16) $$\left\{\begin{array}{l} V_{j}^{\prime }(i)=A_{1j}\cos(2\pi i/N)+B_{1j}\sin(2\pi i/N)+R_{2j}(i)\\ V_{j}^{\prime }(i+m_{2})=A_{1j}\cos(2\pi (i+m_{2})/N)+B_{1j}\sin(2\pi (i+m_{2})/N)+R_{2j}(i+m_{2}) \end{array}\right.$$

When measuring the cross section j, the radial error of the spindle in the measuring coordinate system is $S_{j}(i)$. The data collected by Sensor A and Sensor C are represented by $r_{Aj}^{\prime }(i)$ and $r_{Cj}^{\prime }(i)$, respectively, after eliminating zero-order harmonic components.

According to Equations (8) and (10), Equation (17) can be obtained.

(17) $$\left\{\begin{array}{l} r_{Aj}^{\prime }(i)=V_{j}^{\prime }(i)+S_{j}(i)\cos(2\pi i/N)\\ r_{Cj}^{\prime }(i)=V_{j}^{\prime }(i+m_{2})+S_{j}(i)\cos(2\pi i/N)\cos(\psi )+S_{j}(i)\sin(2\pi i/N)\sin(\psi ) \end{array}\right.$$

By combining Equations (16)–(19), the following can be deduced:

(18) $$r_{Aj}^{\prime }(i)=A_{1j}\cos(2\pi i/N)+B_{1j}\sin(2\pi i/N)+R_{2j}(i)+S_{j}(i)\cos(2\pi i/N)$$

(19) $$\begin{array}{l} r_{Cj}^{\prime }(i)=A_{1j}\cos(2\pi (i+m_{2})/N)+B_{1j}\sin(2\pi (i+m_{2})/N)\\ +R_{2j}(i+m_{2})+S_{j}(i)\cos(2\pi i/N)\cos(\psi )+S_{j}(i)\sin(2\pi i/N)\sin(\psi ) \end{array}$$

Transpose Equation (18) to calculate

(20) $$S_{j}(i)\cos(2\pi i/N)+A_{1j}\cos(2\pi i/N)+B_{1j}\sin(2\pi i/N)=r_{Aj}^{\prime }(i)-R_{2j}(i)$$

Calculate $\mathrm{Equation}\;\left(18\right)\times \cos\psi -\mathrm{Equation}\;\left(19\right)\times 1$ to get

(21) $$\begin{array}{l} \left[r_{Aj}^{\prime }(i)-R_{2j}(i)]\times \cos\psi -[r_{Cj}^{\prime }(i)-R_{2j}(i+m_{2})\right]\\ =[A_{1j}\cos(2\pi i/N)+B_{1j}\sin(2\pi i/N)]\times \cos\psi \\ -A_{1j}\cos[2\pi (i+m_{2})/N]-B_{1j}\sin[2\pi (i+m_{2})/N]\\ +S_{j}(i)\cos(2\pi i/N)\times \cos\psi \\ -[S_{j}(i)\cos(2\pi i/N)\cos\psi +S_{j}(i)\sin(2\pi i/N)\sin\psi ] \end{array}$$

After simplification, we calculate the following:

(22) $$\begin{array}{l} S_{j}(i)\sin(2\pi i/N)-A_{1j}\sin(2\pi i/N)+B_{1j}\cos(2\pi i/N)\\ =\frac{[r_{Cj}^{\prime }(i)-R_{2j}(i+m_{2})]-[r_{Aj}^{\prime }(i)-R_{2j}(i)]\times \cos\psi }{\sin\psi } \end{array}$$

Since the right sides of Equations (20) and (22) are known, they can be represented as $g_{1j}(i)$ and $g_{2j}(i)$, respectively.

(23) $$S_{j}(i)\cos(2\pi i/N)+A_{1j}\cos(2\pi i/N)+B_{1j}\sin(2\pi i/N)=g_{1j}(i)$$

(24) $$S_{j}(i)\sin(2\pi i/N)-A_{1j}\sin(2\pi i/N)+B_{1j}\cos(2\pi i/N)=g_{2j}(i)$$

Calculate $\mathrm{Equation}\;\left(23\right)\times \sin(2\pi i/N)-\mathrm{Equation}\;\left(24\right)\times \cos(2\pi i/N)$ to get

(25) $$\begin{array}{l} {}[S_{j}(i)\cos(2\pi i/N)+A_{1j}\cos(2\pi i/N)+B_{1j}\sin(2\pi i/N)]\sin(2\pi i/N)-\\ \left[S_{j}(i)\sin(2\pi i/N)-A_{1j}\sin(2\pi i/N)+B_{1j}\cos(2\pi i/N)]\cos(2\pi i/N\right)\\ =g_{1j}(i)\sin(2\pi i/N)-g_{2j}(i)\cos(2\pi i/N) \end{array}$$

After simplification, Equation (26) can be obtained.

(26) $$g_{1j}(i)\sin(2\pi i/N)-g_{2j}(i)\cos(2\pi i/N)=A_{1j}\sin(4\pi i/N)-B_{1j}\cos(4\pi i/N)$$

Multiply both sides of Equation (26) by sin(4πi/N), and take the sum in one cycle.

(27) $$\begin{array}{l} \sum_{i=0}^{N-1}\left[g_{1j}(i)\sin(2\pi i/N)-g_{2j}(i)\cos(2\pi i/N)]\sin(4\pi i/N\right)\\ =\sum_{i=0}^{N-1}A_{1j}\sin(4\pi i/N)\sin(4\pi i/N)-\sum_{i=0}^{N-1}B_{1j}\cos(4\pi i/N)\sin(4\pi i/N)\\ =\sum_{i=0}^{N-1}\left[A_{1j}\frac{1-\cos(8\pi i/N)}{2}\right]-\sum_{i=0}^{N-1}\left[B_{1j}\frac{\sin(8\pi i/N)}{2}\right]\\ =\frac{N}{2}A_{1j} \end{array}$$

Obviously, the first-order cosine coefficients can be obtained by transforming Equation (27).

(28) $$A_{1j}=\frac{2\sum_{i=0}^{N-1}\left[g_{1j}(i)\sin(2\pi i/N)-g_{2j}(i)\cos(2\pi i/N)]\sin(4\pi i/N\right)}{N}$$

In the same way, the first-order sine coefficients can be calculated as

(29) $$B_{1j}=-\frac{2\sum_{i=0}^{N-1}\left[g_{1j}(i)\sin(2\pi i/N)-g_{2j}(i)\cos(2\pi i/N)]\cos(4\pi i/N\right)}{N}$$

By this means, $R_{1j}$ can be obtained. According to the principle of the fewest squares, the coordinates of the least squares center of cross section j in the measurement coordinate system $O_{j}-X_{j}Y_{j}Z_{j}$ can be calculated as

(30) $$\left\{\begin{array}{l} x_{j}=\frac{2}{N}\sum_{i=0}^{N}\left[V_{j}^{\prime }(i)\cos(2\pi i/N)\right]=A_{1j}\\ y_{j}=\frac{2}{N}\sum_{i=0}^{N}\left[V_{j}^{\prime }(i)\sin(2\pi i/N)\right]=B_{1j}\\ z_{j}=0 \end{array}\right.$$

Among them, $x_{j}$, $y_{j}$, and $z_{j}$ are the coordinate values of $X_{j}$, $Y_{j}$, and $Z_{j}$ coordinates, respectively.

It should be noted that the installation eccentricity also appears as the first harmonic in the sampled data. Therefore, the position of the least square circle center of the cross section will be affected by the installation eccentricity, which will cause deviation when the profile of each cross section is transformed into the absolute coordinate system. However, since the influence of installation eccentricity on each cross section is equivalent, the influence of installation eccentricity on the position of the cross section can be ignored.

#### 2.3.4. Calculation of $A_{0j}$

By using Equations (23), (24), (28) and (29), the components of the radial error in the $O_{j}X_{j}$ and $O_{j}Y_{j}$ directions can be obtained:

(31) $$\left\{\begin{array}{l} S_{jx}(i)=S_{j}(i)\cos(2\pi i/N)=g_{1j}(i)-A_{1j}\cos(2\pi i/N)-B_{1j}\sin(2\pi i/N)\\ S_{jy}(i)=S_{j}(i)\sin(2\pi i/N)=g_{2j}(i)+A_{1j}\sin(2\pi i/N)-B_{1j}\cos(2\pi i/N) \end{array}\right.$$

By transforming Equation (31), $S_{j}(i)$ can be obtained

(32) $$\begin{array}{l} S_{j}(i)=g_{1j}(i)\cos(2\pi i/N)+g_{2j}(i)\sin(2\pi i/N)\\ -A_{1j}\cos(4\pi i/N)-B_{1j}\sin(4\pi i/N) \end{array}$$

According to Equations (7) and (8), it can be known that

(33) $$\begin{array}{l} r_{Aj}(i)=V_{j}(i)+S_{j}(i)\cos(2\pi i/N)+d_{A}\\ =A_{0j}+A_{1j}\cos(2\pi i/N)+B_{1j}\sin(2\pi i/N)\\ +R_{2j}(i)+S_{j}(i)\cos(2\pi i/N)+d_{A} \end{array}$$

Therefore, the average radius $A_{0j}$ of the cross section can be calculated by using the data collected by Sensor A on one circle

(34) $$\begin{array}{l} A_{0j}=\frac{1}{N}\sum_{i=0}^{N-1}\left[r_{Aj}(i)-A_{1j}\cos(2\pi i/N)-B_{1j}\sin(2\pi i/N)\right]\\ +\frac{1}{N}\sum_{i=0}^{N-1}\left[-R_{2j}(i)-S_{j}(i)\cos(2\pi i/N)\right]-d_{A} \end{array}$$

Considering the existence of zeroing error $d_{A}$, it is equivalent to subtracting a constant from the average radius of each cross section at the same time, so it will not affect the reconstruction of the cylindrical shape.

### 2.4. Cylindrical Shape Reconstruction of High-Precision Mandrel

#### 2.4.1. Coordinate Transformation

In Section 2.3, the calculation algorithms of roundness error, least square circle center, and average radius of each cross section in the measurement coordinate system $O_{j}-X_{j}Y_{j}Z_{j}$ have been introduced. To realistically reconstruct the cylindrical shape of the high-precision mandrel, it is necessary to transform the data of each cross section into the absolute coordinate system.

It is known that the radial error of the spindle in the measurement coordinate system is $S_{j}(i)$, and its components on the coordinate axes $X_{j}$ and $Y_{j}$ are $S_{jx}(i)$ and $S_{jy}(i)$.

(35) $$\left\{\begin{array}{l} S_{jx}(i)=S_{j}(i)\cos(2\pi i/N)\\ S_{jy}(i)=S_{j}(i)\sin(2\pi i/N) \end{array}\right.$$

When the data of cross section j is collected, $S_{ju}(i)$ and $S_{jv}(i)$ are used to represent the components of the spindle radial error on the coordinate axes U and V of the absolute coordinate system, respectively.

The coordinate axis W of the absolute coordinate system is defined as the average line of the spindle rotation axis and therefore

(36) $$\left\{\begin{array}{l} \sum_{i=0}^{N-1}S_{ju}(i)=0\\ \sum_{i=0}^{N-1}S_{jv}(i)=0 \end{array}\right.$$

If the coordinate origin of the measurement coordinate system is $\left(u_{0j},v_{0j},w_{0j}\right)$ in the absolute coordinate system, then

(37) $$\left\{\begin{array}{l} S_{ju}(i)=u_{0j}+S_{jx}(i)\\ S_{jv}(i)=v_{0j}+S_{jy}(i) \end{array}\right.$$

We calculated the left and right sides of Equation (37) separately. In addition, according to the information provided by Equation (36), we can calculate the following:

(38) $$\left\{\begin{array}{l} \sum_{i=0}^{N-1}S_{ju}(i)=\sum_{i=0}^{N-1}\left[u_{0j}+S_{jx}(i)\right]=Nu_{0j}+\sum_{i=0}^{N-1}S_{jx}(i)=0\\ \sum_{i=0}^{N-1}S_{jv}(i)=\sum_{i=0}^{N-1}[v_{0j}+S_{jy}(i)]=Nv_{0j}+\sum_{i=0}^{N-1}S_{jy}(i)=0 \end{array}\right.$$

Therefore,

(39) $$\left\{\begin{array}{l} u_{0j}=\frac{1}{N}\sum_{i=0}^{N-1}S_{jx}(i)\\ v_{0j}=\frac{1}{N}\sum_{i=0}^{N-1}S_{jy}(i)\\ w_{0j}=(j-1)\Delta w \end{array}\right.$$

#### 2.4.2. Reconstruction of Cylindrical Shape of High-Precision Mandrel

The elements of reconstructing the cylindrical shape of the high-precision mandrel have been derived in Section 2.3, and the relationship between the measuring coordinate system and the absolute coordinate system is given in Section 2.4.1. Therefore, the coordinates $\left[u_{j}(i),v_{j}(i),w_{j}(i)\right]$ of each point on the surface of the high-precision mandrel can be determined in the absolute coordinate system, as shown in Equation (40). As a result, the cylindrical shape of the high-precision mandrel is reconstructed.

(40) $$\left\{\begin{array}{l} u_{j}(i)=A_{1j}+u_{0j}+[R_{0}+R_{2j}(i)+A_{0j}]\cos(2\pi ki/N)\\ v_{j}(i)=B_{1j}+v_{0j}+[R_{0}+R_{2j}(i)+A_{0j}]\sin(2\pi ki/N)\\ w_{j}(i)=(j-1)\Delta w \end{array}\right.$$

Among them, $R_{0}$ is the design radius of a high-precision mandrel.

## 3. In Situ Measurement and Reconstruction of Cylindrical Shape

### 3.1. Design of Experimental System

**Figure 5:**
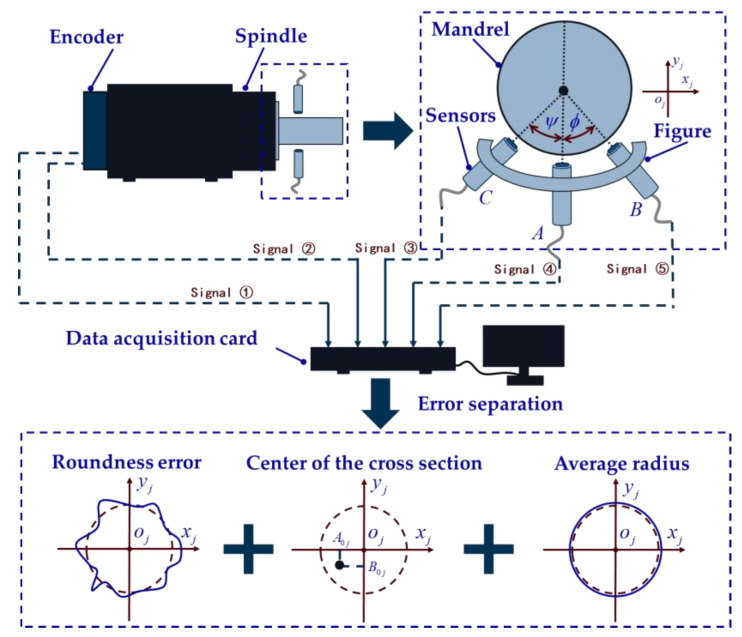
Structural diagram of in situ measurement and reconstruction system.

In the experiment, the measurement system is built based on an ultra-precision lathe, and the structure of the measurement system is shown in Figure 5. The measuring system mainly includes a spindle, encoder, high-precision mandrel, measuring fixture, sensor, data acquisition card, and computer for data processing. The encoder signal type is RS422, and the spindle positioning resolution is 1 arcsec. Three sensors are arranged around the high-precision mandrel at a certain angle to collect data. The data acquisition card collects a total of five sets of data from the encoder and three sensors. The circular grating data are Signal 1 and Signal 2. The data of the three sensors are from Signal 3, Signal 4, and Signal 5, respectively. By using the error separation algorithm written in the computer, the roundness error, the position of the square center of each cross section, and the average radius of each cross section can be separated accurately.

### 3.2. In Situ Measurement and Reconstruction Experiment

**Figure 6:**
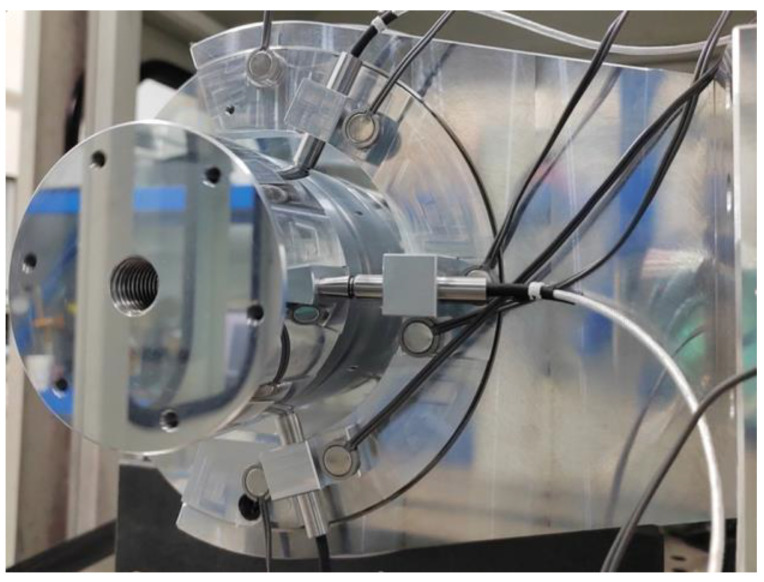
Experimental device.

According to the system structure diagram shown in Figure 5, an experimental device for in situ measurement and reconstruction of a cylindrical shape of a high-precision mandrel is built, as shown in Figure 6.

**Figure 7:**
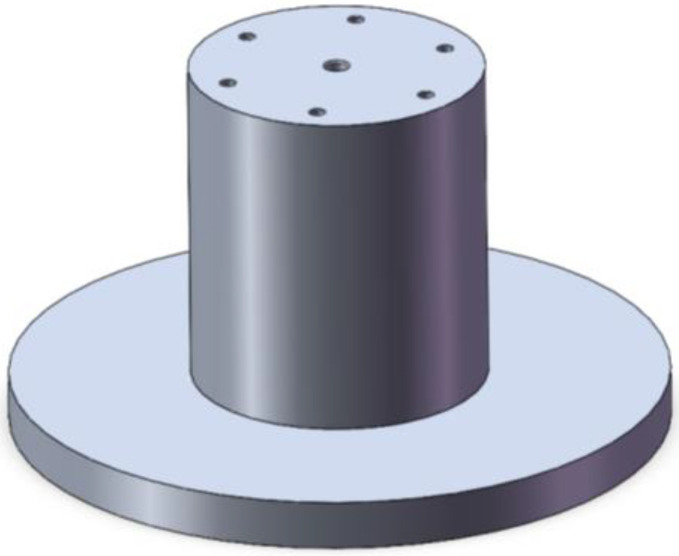
Model of the high-precision mandrel.

The object of in situ measurement is a high-precision mandrel, as shown in Figure 7. The diameter of the mandrel is 90 mm and the length of the mandrel is 100 mm. Through high-precision turning, the roundness of each cross section of the mandrel can be controlled within 0.3 μm while the cylindricity of the mandrel can be kept within 0.5 μm. Since parts with profile accuracy in the range of 0.1–3 μm can be classified as high precision, the mandrel in this study is a high-precision part. Along the axis, a part of the mandrel with a length of 60 mm is selected for measurement. The interval between each cross section is 5 mm, and a total of 13 cross sections are measured. The sensor used in the experiment is the CPL 190/C8-2.0 sensor produced by Lion Precision Company, with a measuring range of 50 μm and a resolution of 1 nm. The sensors chosen for this study are capacitive sensors. The output of the sensors is analog voltage, which can be converted into displacement. The sensor needs to be calibrated before data acquisition to ensure the correctness of its measurement results. In this experiment, the ambient temperature was controlled at 22 ± 0.2 °C.

### 3.3. Measurement Result

**Figure 8:**
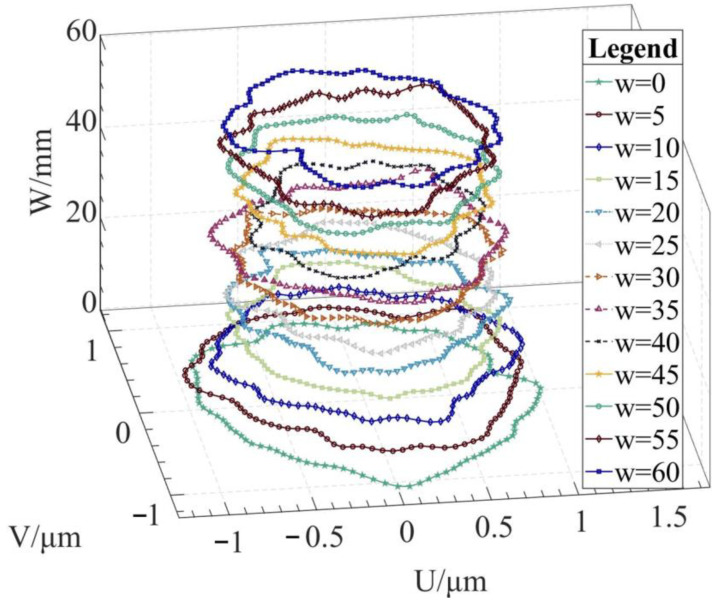
Spatial distribution of each cross section.

**Figure 9:**
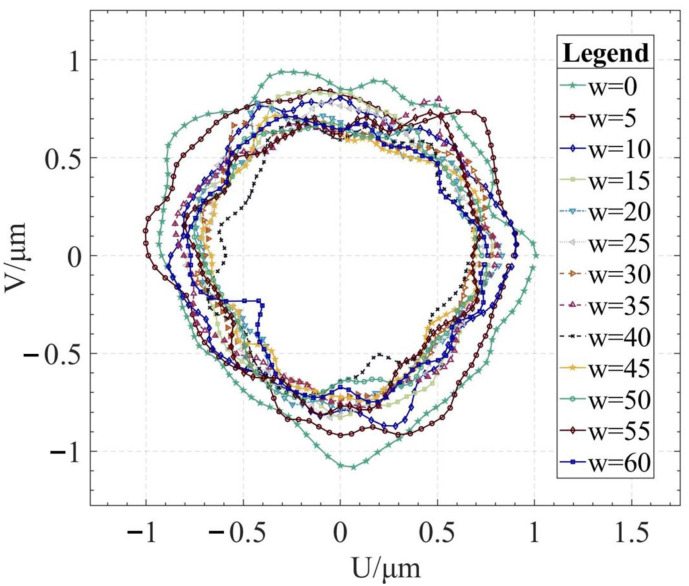
Plane distribution of each cross section.

Using the algorithm in Section 2, the profile data of each cross section can be calculated. The profile data of each cross section in the absolute coordinate system O−UVW is shown in Figure 8 and Figure 9. Figure 8 shows the spatial distribution of each cross section, and Figure 9 shows the distribution of each cross section.

**Figure 10:**
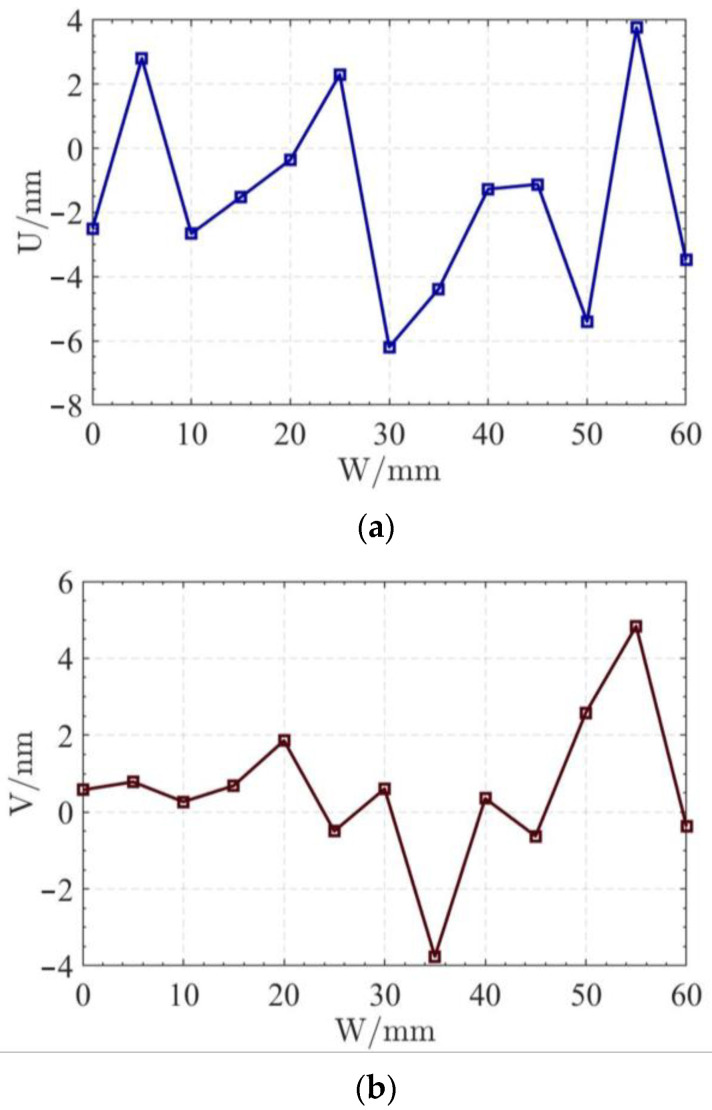

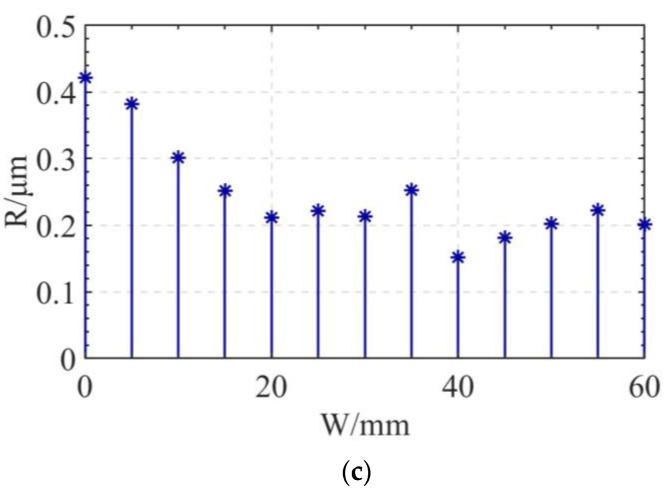
Deviation diagram of least square center and the average radius: (**a**) U coordinates of the least square center of each cross section, (**b**) V coordinates of the least square center of each cross section, and (**c**) Average radius R of each cross section.

In order to display the difference between the least square center position and the radius deviation of each section, the variation curve of the U coordinate of the least square center of each section is shown in Figure 10a. In Figure 10b, the variation curve of the V coordinate of the least square center of each section is shown. In Figure 10c, the variation curve of the average radius of each section is shown.

**Figure 11:**
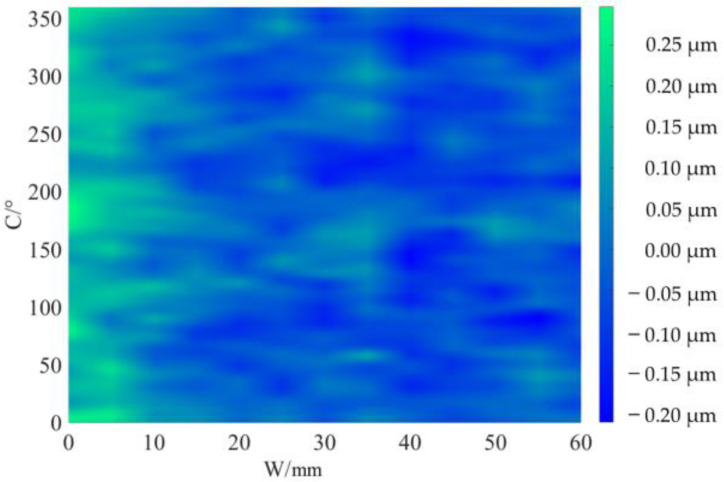
Two-dimensional unfold diagram of cylindrical shape of high-precision mandrel.

**Figure 12:**
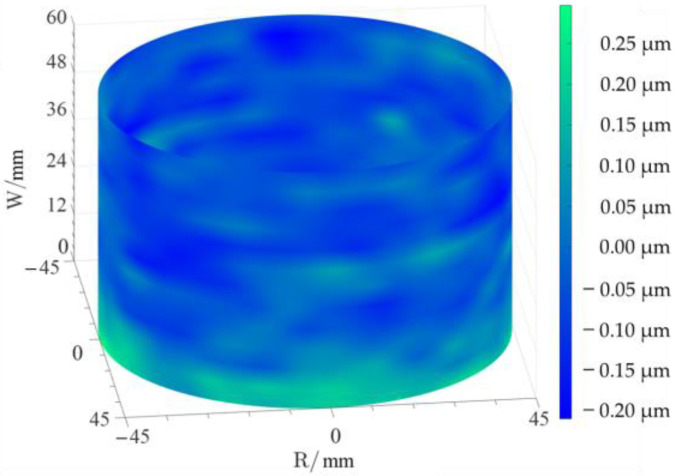
Three-dimensional view of the cylindrical shape.

The profile data of 13 cross sections are used to fit the cylindrical shape, and the fitting results are shown in Figure 11 and Figure 12. Figure 11 is a two-dimensional unfolded diagram of the cylindrical shape, and Figure 12 is a three-dimensional view of the cylindrical shape.

### 3.4. Verification Experiment

**Figure 13:**
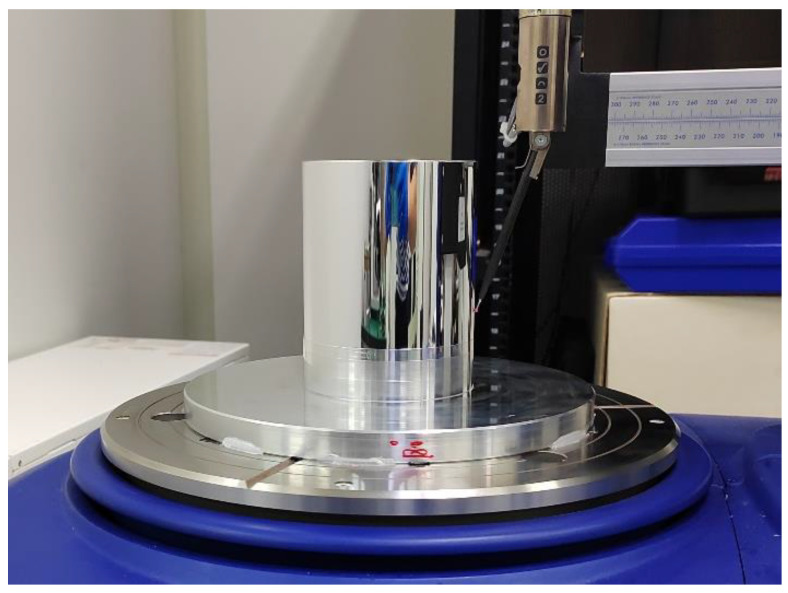
Measurement of high-precision mandrel cylinder using roundness meter.

**Figure 14:**
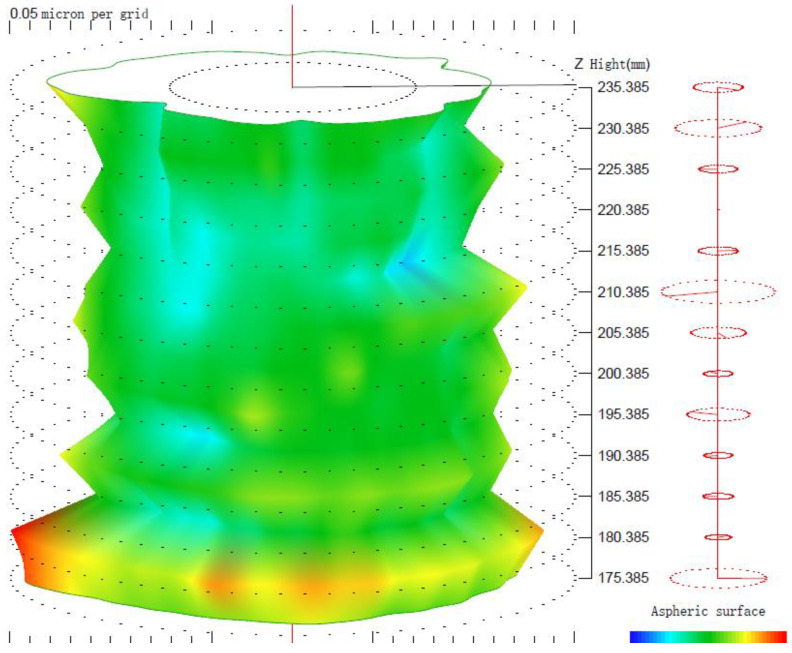
Measurement results of roundness meter.

As shown in Figure 13, the cylindrical shape is verified using a Taylor Hobson roundness meter, and the measured area is consistent with the in situ measurement experiment. The cylindrical shape measured by the roundness meter is shown in Figure 14.

The principle of a roundness meter for measuring the cylindrical shape is also to measure the profiles of multiple sections, and then fit the shape of the cylinder. In this verification experiment, the selected cross sections are the same as those selected in the in situ measurement experiment. The in situ measurement results of four cross sections are selected and compared with those of the roundness meter, as shown in Table 1. All the results of in situ measurement and roundness meter measurement are counted in Table 2.

## 4. Results Analysis and Discussion

### 4.1. Analysis of Experimental Results

The reconstruction method of the cylindrical shape of the high-precision mandrel is to measure the profiles of finite cross sections of the mandrel, and then fit the shape of the high-precision mandrel. Figure 8 and Figure 9, respectively, show the spatial distribution and plane distribution of each cross section measured. We can clearly observe the change in the profile, the relative position, and the difference in the relative radius of each cross section. Figure 10 visually reflects the deviation of the center position of each cross section and the deviation of the average radius of each cross section.

In Table 1, it can be found that the in situ measurement results are consistent with the roundness meter measurement results, but also slightly different. From the profile shape, it can be found that the low-order harmonic components of the in situ measurement and roundness measurement are basically the same, and the differences are mainly high-order harmonic components. In the compensation machining after in situ measurement, the objects of compensation are mainly the low-order harmonic components. Regarding the high-order harmonic components, the main reasons for the differences between in situ measurement and roundness measurement include two aspects: one is the vibration of the machine tool, and the other is the difference in the working principle of the sensors.

**Figure 15:**
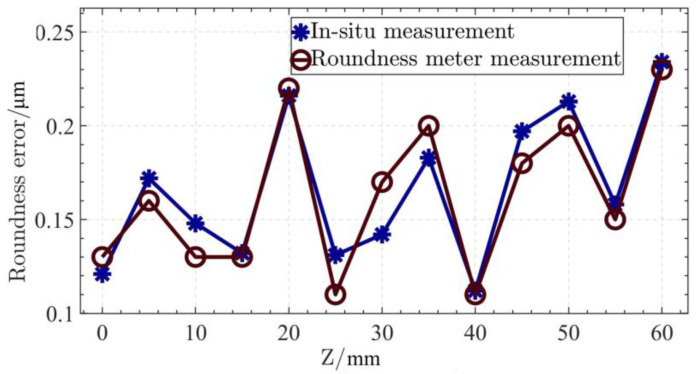
The roundness measurement results of each cross section.

According to Table 2, the in situ measurement results of the roundness of each cross section and that of the roundness meter can be plotted in Figure 15. From Table 2 and Figure 15, it can be known that the in situ measurement results of each cross section are basically the same as those of the roundness meter, and the trend of change is the same. The maximum deviation of roundness of each cross section is 30 nm, which is about 17.65% of the measurement result of the commercial roundness meter. Considering the influence of the measurement environment, the measurement uncertainty of the in situ measurement system is within the acceptable range. By averaging multiple measurements, the accuracy of measurement results can be ensured.

The cylindrical shape measured by the in situ measurement and reconstruction technology of the cylindrical shape of the high-precision mandrel by the three-point method of the high-precision mandrel is shown in Figure 11 and Figure 12. The cylindrical shape measured by the roundness meter is shown in Figure 14. By comparing the measurement results in Figure 12 and Figure 14, it can be observed that the in situ measurement and reconstruction result of the cylindrical shape is consistent with the measurement result of the roundness meter. At the same time, according to the measurement results of cylindricity in Table 2, it can be found that the deviation of cylindricity measurement results is 10 nm, which is 2.56% of the measurement result of the commercial roundness meter. Therefore, the in situ measurement and reconstruction technology of the cylindrical shape of the high-precision mandrel using the three-point method can be proven to be accurate.

### 4.2. Analysis of Measurement Uncertainty

According to the algorithm given in Section 2 and the experimental results in Section 3, it can be found that both the radial error of the spindle and the zeroing error of the sensor are effectively separated theoretically and experimentally. However, what cannot be ignored in the measurement is that the installation error of the sensors will also affect the measurement result. Since a high-precision fixture was used to fix multiple sensors in this study, the high accuracy of the fixture allows for accurate sensor mounting positions. At the same time, based on the working principle and performance of capacitive sensors, the effect of small errors in sensor installation on the measurement results can be ignored.

The environmental condition is another important factor affecting measurement accuracy. This study avoids the influence by strictly controlling the environmental factors. By building an independent, closed experimental space, the interference of external noise and vibration is avoided. The temperature of the experimental environment is controlled at 22 ± 0.2 °C by using a temperature control system. During the measurement process, the mandrel and the measurement equipment are grounded to avoid electrostatic interference. Meanwhile, the reliability of the measurement data is ensured due to the strong environmental adaptability of the capacitive sensors.

### 4.3. Advantages of the In Situ Measurement and Reconstruction Technology

Compared with the roundness meter, the in situ measurement and reconstruction technology of the cylindrical shape of the high-precision mandrel using the three-point method has great advantages. Not only can the secondary clamping error be avoided, but also the efficiency of measurement and compensation machining can be improved. The cost of in situ measurements is lower compared to off-position measurements. For example, the cost of purchasing a roundness meter with proper accuracy is about USD 100,000–150,000, while building an in situ measurement system is only 1/15 of the cost of purchasing a roundness meter. The relative cost of in situ measurements will be even lower if the time cost saved by the efficiency of in situ measurements is taken into account. Therefore, the manufacturing cost of the high-precision mandrel can be effectively reduced by applying in situ measurement and reconstruction technology.

The technology proposed in this paper can accurately identify the straightness of the high-precision mandrel axis, while the roundness meter cannot realize the measurement of the straightness of the axis. This means that better results can be achieved by using the measurement results of the technology proposed in this paper as the basis of compensation machining.

In addition, the technology proposed in this paper optimizes the calculation method of the average radius of the cross section, avoiding the interference of the sensor zeroing error. Compared with other multi-point measurement methods, the technology proposed in this paper has a simpler measuring device and fewer error components, so it is more suitable for in situ measurement and cylindrical shape reconstruction of small-sized and high-precision mandrels.

## 5. Conclusions

In this paper, the three-point method for cylindricity measurement is optimized, and the in situ measurement and reconstruction technology of the cylindrical shape of a high-precision mandrel using the three-point method is proposed. Theoretical analysis and experimental verification are given in detail in this paper, and an in situ measurement and reconstruction system is designed. The correctness of the measurement results of the system is verified by using a commercial roundness meter, and the deviation of cylindricity measurement results is 10 nm, which is 2.56% of the measurement result of a commercial roundness meter. Theoretical analysis and experimental results show that the in situ measurement and reconstruction technology proposed in this paper can greatly improve the efficiency and accuracy of compensation machining. The research content of this paper can be used for reference to promote the development of in situ measurement and compensation machining technology of high-precision mandrels, such as mandrels for the manufacture of ultra-precision spindles and mandrels for X-ray grazing incidence mirror replication. The in situ measurement system designed in this paper is independent of the machine tool and the structure is complex, which cannot realize the measurement of the whole mandrel. At the same time, the uncertainty of in situ measurement is less analyzed due to the limitation of the length of the paper. Simplifying the measurement system and integrating it into the machine tool will be the future research direction of this study.

## Figures and Tables

**Table 1:** Comparison of measurement results of cross sections.

Serial Number	In Situ Measurement	Roundness Meter
1	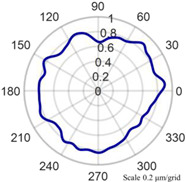	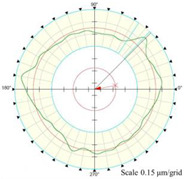
2	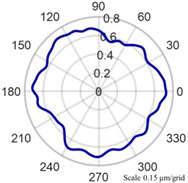	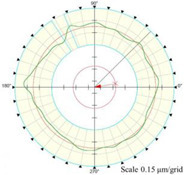
3	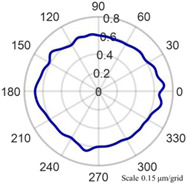	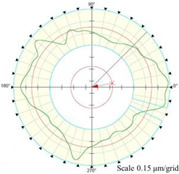
4	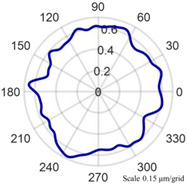	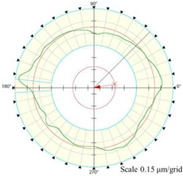

**Table 2:** Statistics of results of in situ measurement and roundness meter measurement.

Measurement Content		In Situ Measurement/μm	Roundness Meter/μm	Deviation/μm
Cylindricity		0.40	0.39	0.01
Roundness of cross section	1	0.12	0.13	0.01
	2	0.17	0.16	0.01
	3	0.15	0.13	0.02
	4	0.13	0.13	0.00
	5	0.22	0.22	0.00
	6	0.13	0.11	0.02
	7	0.14	0.17	0.03
	8	0.18	0.20	0.02
	9	0.11	0.11	0.00
	10	0.20	0.18	0.02
	11	0.21	0.20	0.01
	12	0.16	0.15	0.01
	13	0.23	0.23	0.00

## Data Availability

The data presented in this study are available upon request from the corresponding author. The data are not publicly available because it is part of an ongoing study.
